# Addition of cyclophosphamide and higher doses of dexamethasone do not improve outcomes of patients with AL amyloidosis treated with bortezomib

**DOI:** 10.1038/bcj.2017.47

**Published:** 2017-06-16

**Authors:** E Kastritis, M Gavriatopoulou, M Roussou, D Fotiou, D C Ziogas, M Migkou, E Eleutherakis-Papaiakovou, I Panagiotidis, N Kanellias, E Psimenou, E Papadopoulou, C Pamboucas, E Manios, H Gakiopoulou, A Ntalianis, A Tasidou, S Giannouli, E Terpos, M A Dimopoulos

**Affiliations:** 1 Department of Clinical Therapeutics, National and Kapodistrian University of Athens, School of Medicine, National and Kapodistrian University of Athens, Athens, Greece

## Abstract

Bortezomib, in combination with dexamethasone (VD) or with the addition of cyclophosphamide (VCD), is highly effective in patients with amyloid light-chain (AL) amyloidosis. Currently, VCD is considered as a primary regimen for patients with AL, but it is not clear whether the addition of cyclophosphamide to VD further and significantly improves efficacy, given the substantial activity of bortezomib itself. We retrospectively compared the outcomes of 101 patients with AL amyloidosis who received VD (*n*=59) or VCD (*n*=42) in two consecutive periods. Early mortality after adjustment for Mayo stage was similar. On intent to treat, a hematologic response rate was 68% for patients treated with VD and 78% for VCD (*P*=0.26), while complete response+very good partial response (CR+VGPR) rate was 47.5% and 35%, respectively. Higher doses of dexamethasone or twice-weekly bortezomib were not associated with significantly higher CR+VGPR rates. Organ responses occurred in similar rates between the two groups. Median survival was similar (33 vs 36 months, *P*=0.45) even after adjustment for Mayo stage and dose and schedule of bortezomib and dexamethasone. In conclusion, bortezomib even with low doses of dexamethasone is effective for the treatment of AL amyloidosis; higher doses of dexamethasone and addition of cyclophosphamide do not seem to have a profound effect on efficacy and survival.

## Introduction

Treatment of amyloid light-chain (AL) amyloidosis is based on the elimination of the plasma cell clone that produces the amyloidogenic light chains.^1^ Typically, the plasma cell clone in AL amyloidosis is indolent and the plasma cell burden is low;^1, 2, 3^ thus, even low-dose, low-toxicity, regimens may be effective and may induce complete hematologic responses in a significant proportion of patients.^4^ Bortezomib is a very effective drug in targeting plasma cells and can rapidly induce plasma cell apoptosis. In the clinical setting, several lines of data have shown that bortezomib either as single agent^5^ or in combinations, with dexamethasone (VD)^6, 7^ or with the addition of cyclophosphamide (VCD),^8, 9, 10^ induces high rates of hematologic complete responses and organ responses. Dexamethasone has rapid antiplasma cell activity and pulsed dexamethasone^11^ or concomitantly with alkylating agents^12^ or bortezomib is commonly used. However, dexamethasone may be poorly tolerated by patients with AL amyloidosis^6, 11^ because of the frailty associated with the multisystemic amyloidotic involvement and poor tolerance of steroids’ effects in cardiovascular and other systems. Thus, treatment combinations for patients with AL amyloidosis should provide efficacy but with the lowest toxicity.

In elderly frail patients with myeloma, many of which have significant comorbidities, prospective randomized data indicate that the addition of a third agent to VD does not improve outcomes while it may increase toxicity.^13, 14^ For patients with AL amyloidosis, the combination of VCD is considered as a ‘standard’ regimen for primary therapy in most centers,^1, 15, 16^ but it is not clear whether the addition of a third drug (cyclophosphamide) to the bortezomib/dexamethasone (VD) backbone further and significantly improves efficacy, given the substantial activity of bortezomib itself. Also, it is not clear whether cyclophosphamide is the optimal partner of VD, among other potential partners, including other alkylating agents.

The aim of this analysis was to compare the outcomes of patients with AL amyloidosis who have received primary therapy with VD to that of patients who received VCD to evaluate the incremental value of the addition of cyclophosphamide to VD and the role of dexamethasone doses.

## Patients and methods

The analysis included 101 consecutive patients with biopsy-confirmed AL amyloidosis, all of which were diagnosed and treated in the Department of Clinical Therapeutics, Athens, Greece. All patients received similar supportive care and were treated in two consecutive periods: all patients from 2005 up to 2010 received VD, and after January 2011 and until 2013, VCD was given in all patients.

All patients had biopsy-confirmed diagnosis of AL amyloidosis. Patients with localized amyloidosis or amyloid other than AL were not eligible. Organ involvement, response and progression was defined based on the 2005 criteria^17^ and the modified criteria proposed for heart and renal involvement and response evaluation.^18, 19^ Serum-free light chains were measured by nephelometry using Freelite Serum-Free Light Chain Assays (The Binding Site, San Diego, CA, USA). Glomerular filtration rate was estimated glomerular filtration rate according to Kidney Disease Outcomes Quality Initiative guidelines using the Modification of Diet in Renal Disease formula (estimated glomerular filtration rate estimated according to serum creatinine). Hematologic response was assessed according to the 2012 criteria.^18^ Data were collected prospectively in all patients, and all were assessed and followed rigorously according to a prespecified institutional protocol and received similar supportive care according to our institution’s practice. Assessment for hematologic response is performed monthly. Patients have given informed consent for collection and analysis of data. This study was approved by the institutional review board of ‘Alexandra’ hospital (Scientific Committee of ‘Alexandra’ Hospital).

### Treatment

Bortezomib was given initially as an intravenous bolus infusion, but, after 2011, it was given subcutaneously. According to our institutional policy, bortezomib schedule (weekly vs two times per week) was based on the risk profile of the patients, mainly cardiobiomarker stage (Mayo stage^20^). Dexamethasone dose was also risk adapted.^21^ The dose of dexamethasone for the purpose of this analysis was calculated as total dose per month in mg (mg per month) and not per cycle. Also, the use of pulsed dexamethasone (i.e. 4 consecutive days) vs non-pulsed (i.e. weekly or two times per week) was also recorded. Cyclophosphamide was given as an intravenous infusion at a dose of 300 mg/m^2^ and at a maximum of 500 mg weekly for 3 consecutive weeks.

### Statistical analysis

All efficacy analyses are on intent-to-treat basis. Time to event (progression, death, response) was calculated from the date of first treatment until the date progression, death or other event or until the date of last follow-up, if the respective event has not occurred. Cox models were used to compute hazard ratios (HRs). Multivariate analysis was performed using a proportional hazard model. *P*-values <0.05 were deemed statistically significant; all tests were two sided. Analyses were performed using R software (R Core Team (2013), http://www.R-project.org/) and SPSS (IBM SPSS Statistics for Windows, version 20; IBM Corp., Armonk, NY, USA).

## Results

The median age of all patients in the analysis was 65 years, 70% had cardiac and 71% renal involvement; the Mayo stage was 1, 2 and 3 in 20%, 47% and 33%, while the renal stage was 1, 2 and 3 in 22%, 56% and 22% of the patients, respectively.

Treatment given was VD in 59 (58%) and VCD in 42 (42%) patients. As mentioned, patients were not matched but were treated in two consecutive periods. Thus, compared with patients who received VCD, those patients who received VD were older (median age 67 vs 60.5 years, *P*=0.03), had more often Mayo stage 3 disease (42% vs 22%, *P*=0.026), had lower estimated glomerular filtration rate (median 54 vs 86 ml/min per 1.73 m^2^, *P*=0.021) and thus less patients were renal stage 1;^19^ however, renal stage 3 patients were similar. Heart, renal and nerve involvement were similar between those who received VD vs VCD (Table 1). Weekly bortezomib was given in 41% of patients who received VD and in 40% of those treated with VCD. The starting dose was 1.3 mg/m^2^ in 90 and 92.5% of patients, respectively. The median dose of dexamethasone was 240 mg per month for patients treated with VD and was 144 mg per month for those treated with VCD (*P*=0.01). Pulsed dexamethasone was given in 41% of the patients treated with VD.

### Patient outcomes after therapy

On intent to treat, a hematologic response was achieved by 72% of all patients (CR: 25% VGPR: 17% partial response (PR): 30%): it was 68% for patients treated with VD and was 78% for VCD (*P*=0.26) (Table 2). Hematologic responses were not different between the two groups for patients of different Mayo stages (83% vs 86% for stage 1, 68% vs 87% for stage 2 and 60% vs 57% for stage 3). After adjustment for Mayo stage, there was still no difference in response rates. Regarding CR+VGPR rates, it was 47.5% with VD and 35% with VCD (*P*=0.185). Time to first hematologic response (at least hemPR) was similar, 1.2 and 1.3 months, respectively, for VD- and VCD-treated patients (*P*=0.85); median time to ⩾VGPR was also similar (1.8 vs 1.9 months, *P*=0.9).

Higher doses of dexamethasone or twice-weekly bortezomib schedule were not associated with significantly higher hematologic response rates or CR+VGPR rates: 46% vs 36% of patients treated with twice weekly vs once weekly achieved CR+VGPR, respectively (*P*=0.414).

The use of pulsed dexamethasone was not associated with higher rates or better quality of hematologic responses (29% vs 36.5% for non-pulsed dexamethasone, *P*=0.3). Twice-weekly bortezomib was associated with more rapid response (median time to first response, i.e. ⩾PR, was 1 vs 1.6 months for weekly bortezomib), but it was not statistically significant (*P*=0.472), whereas higher doses of dexamethasone or pulsed dexamethasone did not induce more rapid responses (median time to first response was 1.4 vs 1 month for non-pulsed dexamethasone, *P*=0.474).

Organ responses were recorded in 35% of patients (cardiac in 26%, renal in 42%). For VD-treated patients, cardiac response rate was 29% and renal response rate was 43%, whereas for VCD-treated patients cardiac response rate was 21% and renal response rate was 41% (*P*>0.5 for all comparisons).

**Figure 1:**
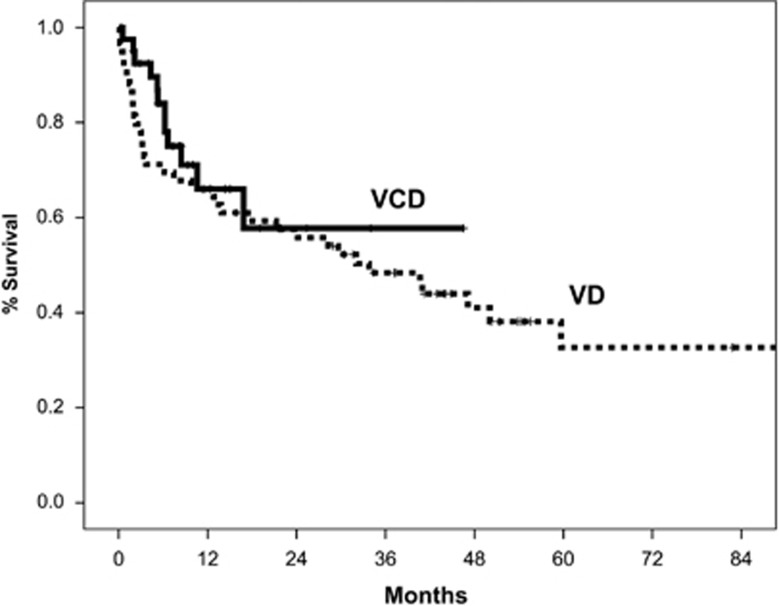
OS of patients treated with VD or VCD (all patients).

**Figure 2:**
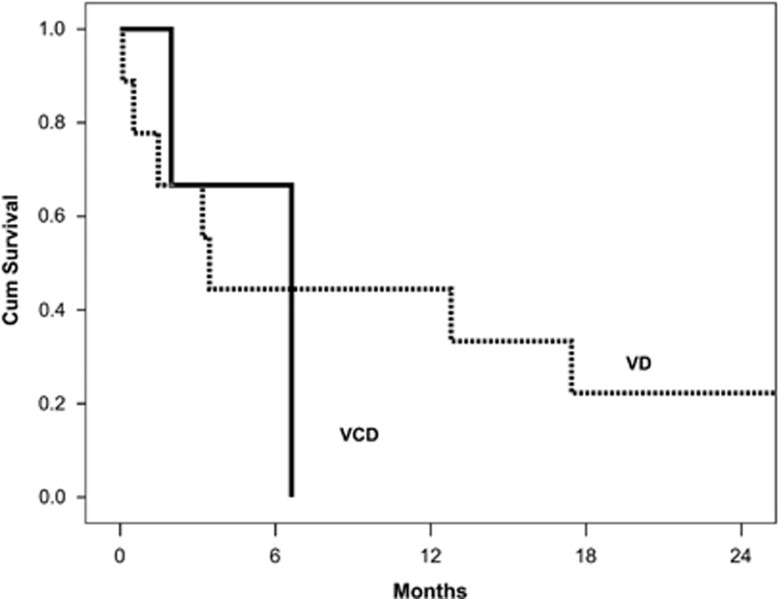
OS of patients with stage 3B disease treated with VD or VCD.

Median follow-up for all patients is 3 years and the median overall survival (OS) is 34 months. Median OS of patients treated with VD vs VCD was similar (33 vs 36 months, *P*=0.45) (Figure 1). Early mortality (within <3 months from the start of therapy) was 22% for patients treated with VD and 8% for patients treated with VCD. However, after adjustment for Mayo stage there was no difference, and was 36% vs 29% among patients with Mayo stage 3 disease. The 3-year OS for patients treated with VD vs VCD at each Mayo stage was 92% vs 100% for stage 1, 59% vs 69% for stage 2 and 40% vs 45% for stage 3, respectively (Supplementary Figure S1). There was also no difference between the two groups among patients with stage 3B disease (Figure 2).

After adjustment for the dose and schedule of bortezomib and dexamethasone, and Mayo stage, there was still no difference in the OS between patients treated with VD or VCD in the multivariate analysis (Table 3). Furthermore, no prognostic effect of higher doses of dexamethasone and twice-weekly bortezomib was found in multivariate analysis.

### Toxicity

Hematologic toxicity was minimal even with the addition of cyclophosphamide to VD and there was no difference in grade 3 or 4 cytopenias, which occurred in <10% in both groups. Neuropathy rates were similar (any grade neuropathy 61% vs 55%) and were somewhat lower in patients treated with subcutaneous bortezomib compared with those treated with intravenous bortezomib, so that grade 2 and 3 neuropathy was more common in patients treated with VD, which mostly received intravenous bortezomib (14% vs 7% for VCD). Fluid retention was also more common in patients treated with VD, probably due to the more common use of pulsed dexamethasone. Overall 72% of patients treated with VD and 80% of patients treated with VCD required dose reductions of bortezomib, mostly for neuropathy and fatigue. No patient discontinued cyclophosphamide because of toxicity.

## Discussion

In this retrospective analysis, we observed that the addition of cyclophosphamide and higher doses of dexamethasone did not provide any substantial incremental efficacy to the ‘backbone’ of bortezomib and dexamethasone in patients with newly diagnosed, previously untreated, AL amyloidosis. These results indicate that bortezomib is very effective and that the addition of more than two drugs and higher doses may not only increase the efficacy further but also raise the question as to which may be the best partner to increase bortezomib efficacy.

Our results are not surprising. Two prospective randomized studies in elderly frail myeloma patients have also shown that adding either cyclophosphamide or melphalan or thalidomide to bortezomib and dexamethasone does not offer significant benefit in terms of response rates and progression-free survival, although it increases toxicity.^13, 14^ Triple combinations are considered the standard for induction in younger, transplant eligible myeloma patients,^22^ but our data and the data from elderly frail myeloma patients show that it is difficult to extrapolate the results from studies in fitter patients and apply them in frail patients, such as those with AL amyloidosis.

There are other retrospective comparisons of triplet vs doublet combinations in patients with AL amyloidosis.^10, 23^ However, these studies have compared bortezomib- with non-bortezomib-containing regimens. The prospective study comparing BMDex with MDex also compares a bortezomib-containing with a non-bortezomib-containing regimen; thus, our study is quite different in this respect.

Nevertheless, there are some limitations in our study. This is a retrospective analysis and although the patients were treated in consecutive periods reducing selection bias, they were not matched for major characteristics. It is, however, notable that patients treated with VD had more high-risk features than VCD-treated patients. Salvage regimens were not different between the two cohorts. The power of this study to detect a real difference in the outcomes of those treated with VCD vs VD is also limited. The duration of follow-up in the VCD group is shorter than that in the VD group and could affect the evaluation of organ responses, as in some patients organ response may require prolonged periods to become evident.^24^ Another important question concerns the dose of cyclophosphamide, which is lower than what is commonly used in myeloma patients. Such moderate doses are commonly used in most centers,^8, 9, 10^ although some physicians prefer to use oral rather than the intravenous route, which may affect pharmacodynamics and pharmacokinetics of cyclophosphamide as this drug needs to be activated in the liver. Nonetheless, hematologic response rates in our patients are very similar to those reported in larger collaborative series.^9^ Higher doses of cyclophosphamide could probably be more effective, as in the EVOLUTION study, but at the expense of increased toxicity.^25^ Recent data indicate that patients bearing plasma cell clones with certain cytogenetic abnormalities may have a less favorable outcome with bortezomib-based therapies;^26^ however, cytogenetics were available only in a small number of patients, thus we cannot make statistically meaningful comparisons.

Since the addition of cyclophosphamide and higher doses of dexamethasone may not offer a substantial benefit, which drug could be a better partner for bortezomib? Melphalan has also been used, as in the combination of bortezomib with MDex. The results of the prospective randomized study comparing BMdex with MDex are to be presented, but a retrospective comparison^23^ has shown that the efficacy of BMDex is not very different from that reported with VCD.^9^ Probably drugs with non-overlapping toxicity and new mechanisms may be better partners for bortezomib. In this regard, a non-neurotoxic IMiD, such as lenalidomide, could be a candidate, and VRD combination has been used in young newly diagnosed and in fit elderly myeloma patients.^27^ However, lenalidomide may not be well tolerated by patients with AL amyloidosis and lower doses need to be used.^28, 29, 30, 31, 32^ Another attractive partner for bortezomib could be a monoclonal antibody such as daratumumab. In the CASTOR study, the combination of daratumumab with VD showed impressive efficacy in patients with relapsed myeloma.^33^ Although data are limited, daratumumab was active in patients with refractory AL amyloidosis.^34^

In conclusion, our data indicate that bortezomib even with low doses of dexamethasone is effective for the treatment of AL amyloidosis. Higher doses of dexamethasone and addition of a third agent (cyclophosphamide) does not seem to have a profound effect on the efficacy of bortezomib combinations. Our data also indicate the limits of bortezomib-based therapies, and new agents either targeting the plasma cell clone (like monoclonal anti-CD38) or targeting the amyloid deposits are needed.

## Figures and Tables

**Table 1:** Characteristics of the patients in the analysis

	*VD (*N=*59)*	*VCD (*N=*42)*	P*-value*
Age (years)	67	60.5	0.03
Heart	41 (69.5%)	30 (71%)	0.894
Renal	44 (75%)	28 (66%)	0.344
Peripheral nerve	15 (25%)	7 (17%)	0.201
*Bone marrow plasma cell infiltration (median/IQR)*	*15% (8–20%)*	*15% (8–20%)*	*0.782*
Mayo stage 1	12 (20%)	8 (18%)	
Mayo stage 2	22 (37%)	25 (60%)	0.026
Mayo stage 3	25 (42%)	9 (22%)	
Mayo stage 3b	10 (17%)	4 (10%)	0.2
			
dFLC (mg/l) (median/IQR)	270 (75–569)	237 (76–715)	0.927
Revised Mayo stage 1	12 (20%)	11 (26%)	
Revised Mayo stage 2	15 (25%)	8 (20%)	0.324
Revised Mayo stage 3	14 (24%)	16 (37%)	
Revised Mayo stage 4	18 (31%)	7 (17%)	
			
eGFR ml/min per 1.73 m^2^ (median/IQR)	54 (23–99)	86 (50–109)	0.021
Renal stage 1	12 (27%)	14 (50%)	
Renal stage 2	25 (57%)	10 (35%)	0.054
Renal stage 3	7 (16%)	4 (15%)	
Dexamethasone per month (mg) (median/IQR)	240 (72–240)	144 (72–160)	0.01
Bortezomib weekly	24 (41%)	17 (40%)	0.814
Bortezomib intravenous	54 (88%)	5 (12%)	<0.001
Second-line therapy	17 (29%)	10 (24%)	0.556
Lenalidomide	6 (10%)	6 (14%)	
MDex	7 (12%)	3 (7%)	0.666
Bortezomib	3 (5%)	1 (2%)	
Other	1 (2%)	0	

Abbreviations: dFLC, differential serum-free light; eGFR, estimated glomerular filtration rate; IQR, interquartile range; MDex, melphalan plus high-dose dexamethasone; VCD, cyclophosphamide; VD, dexamethasone.

**Table 2:** Outcomes of patients treated with VD vs VCD (intent to treat)

	*VD*	*VCD*	P*-value*
Overall hematologic response	68%	78%	0.26
CR	27%	21%	0.514
CR+VGPR	47.5%	35%	0.185
Time to first hematologic response (months)	1.2	1.3	0.85
Renal response	43%	41%	0.774
Cardiac response	29%	21%	0.519

Abbreviations: CR, complete response; VCD, cyclophosphamide; VD, dexamethasone; VGPR, very good partial response.

**Table 3:** Multivariate analysis of factor associated with overall survival

	P*-value*	*HR*	*95.0% CI for HR*	
			*Lower*	*Upper*
Doublet vs triplet (VD vs VCD)	0.638	0.831	0.354	1.685
Revised Mayo stage 1		1		
Revised Mayo stage 2	**0.06**	3.6	0.945	13.97
Revised Mayo stage 3	**0.003**	6.7	1.902	23.72
Revised Mayo stage 4	**0.001**	9.3	2.469	35.64
Dexamethasone ⩽160 mg per month	0.312	2.809	0.380	20.772
Pulsed dexamethasone	0.469	0.578	0.131	2.551
Bortezomib doses per week (1 vs 2)	0.359	0.492	0.108	2.243

Abbreviations: CI, confidence interval; HR, hazard ratio; VD, dexamethasone; VCD, cyclophosphamide. Bold number indicate that are statistically significant.
