# Supplementary material for: Addition of cyclophosphamide and higher doses of dexamethasone do not improve outcomes of patients with AL amyloidosis treated with bortezomib
Source: Blood Cancer J. 2017 Jun 16;7(6):e570–. doi: 10.1038/bcj.2017.47 (PMC5520394; doi:10.1038/bcj.2017.47)

Figure S1: overall survival in diffrenet Mayo stages for patients treated with VD (blue line) vs VCD (green line)


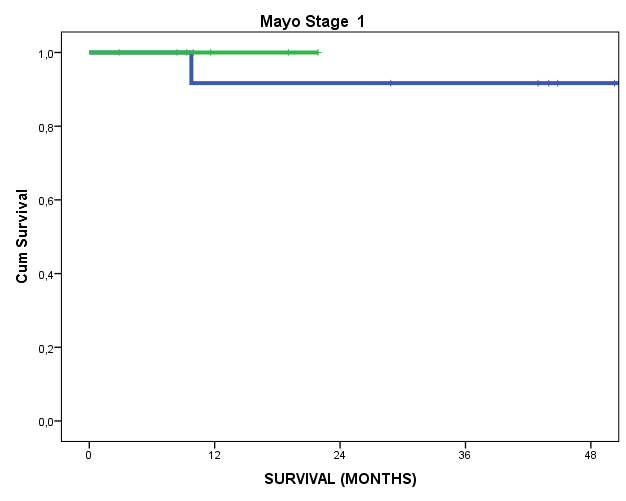


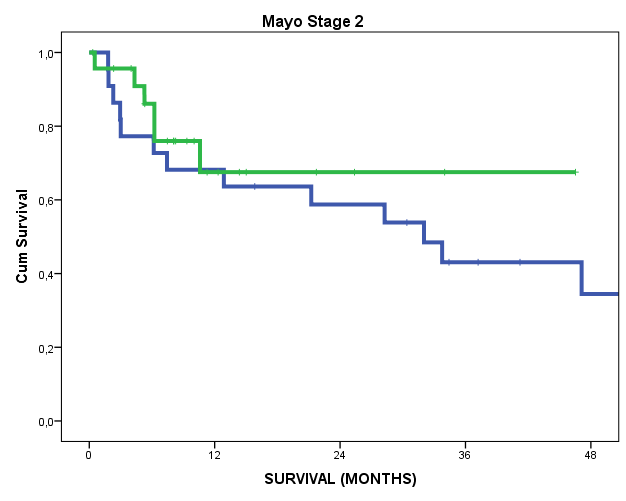


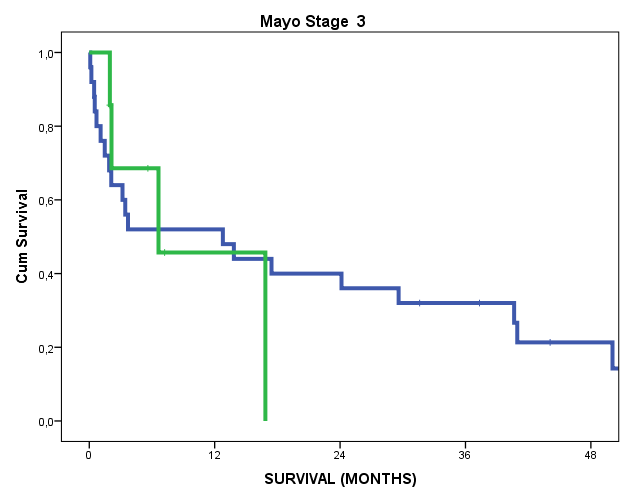

Supplement: Supplementary Figure [file bcj201747x1.docx]
